# Impacts of heavy smoking on non-coding RNA expression for patients with esophageal carcinoma

**DOI:** 10.1186/s12920-023-01574-z

**Published:** 2023-07-05

**Authors:** Qin Wen, Xinlan Mao, Xinling Shi, Yuting Wang, Jianming Wang

**Affiliations:** Department of Epidemiology, Center for Global Health, School of Public Health, Nanjing Medical University, Nanjing, 211166 China

**Keywords:** Tobacco smoking, Non-coding RNA, RNA regulatory network, Immune infiltration, Esophageal carcinoma, Prognosis

## Abstract

**Background:**

Smoking is a well-recognized risk factor for esophageal carcinoma, but the underlying molecular mechanism remains unclear. Previous studies have demonstrated the predictive value of non-coding RNA (ncRNA) for the prognosis of esophageal carcinoma; however, the expression of smoking-related ncRNAs has not been systematically characterized. Herein, we comprehensively assessed the hazard of heavy smoking and its impact on ncRNA expression patterns in patients with esophageal carcinoma.

**Methods:**

Transcriptome and clinical features of patients with esophageal carcinoma were acquired from The Cancer Genome Atlas (TCGA) database. Cox regression analysis was employed to calculate the hazard ratio (HR) of smoking behavior. Differential expression analysis was conducted with the “edgeR” package. The smoking-related RNA regulatory network was based on lncRNA‒miRNA and miRNA‒mRNA pairs and visualized by Cytoscape 3.7.1. We applied Gene Ontology (GO) and Kyoto Encyclopedia of Genes and Genomes (KEGG) pathway enrichment analyses for functional annotation. Univariate and least absolute shrinkage and selection operator (LASSO) Cox regression analyses were used for model construction. We applied Kaplan‒Meier analysis with a log-rank test for survival analysis, with group comparison by the Wilcoxon signed ranked test.

**Results:**

Heavy smoking contributed to the poor overall survival of esophageal carcinoma, with an HR of 3.167 (95% CI: 1.077–9.312). A total of 195 lncRNAs and 73 miRNAs were differentially expressed between patients with or without smoking behavior. We constructed smoking-related RNA regulatory networks, and functional annotation enriched a series of cancer-related pathways. We generated a smoking-related prognostic risk score and found that patients with a high score had a poor prognosis. Fourteen out of 23 immune cell types differentially infiltrated into a distinct risk group, while no correlation was observed between the risk score and immune cells.

**Conclusion:**

Altogether, we profiled smoking-related ncRNA expression patterns and constructed an RNA regulatory network, providing a landscape of smoking-related molecular mechanisms of esophageal carcinoma. The smoking-related risk score, which was related to prognosis, revealed that tobacco smoking could suppress tumor immunity via the ncRNA mechanism.

**Supplementary Information:**

The online version contains supplementary material available at 10.1186/s12920-023-01574-z.

## Introduction

Esophageal carcinoma, with approximately 604,000 new cases and 544,000 deaths in 2020, remains the seventh most common cancer and the sixth leading cause of cancer-related death worldwide [1]. Epidemiological studies have demonstrated that the geographic variation in esophageal carcinoma incidence substantially differs between esophageal squamous cell carcinoma (ESCC) and esophageal adenocarcinoma (EAC) [2]. Over the past decades, the incidence of ESCC has been broadly declining in some high-risk regions in Asia, whereas the incidence of EAC has significantly risen in developed countries. Although ESCC and EAC share overwhelmingly distinct etiologies, tobacco smoking is a well-established common factor [3]. Observational data suggested that active smoking contributes to an over fivefold increased risk of ESCC [4, 5] and a nearly threefold risk of EAC. Although polycyclic hydrocarbons, nitrosamines, and acetaldehyde are common harmful substances, the underlying molecular mechanism induced by smoking remains unclear. Over the past decades, significant efforts have been dedicated to screening strategies, diagnostic approaches, and therapeutic regimens in esophageal carcinoma, but the prognosis remains unsatisfactory. Importantly, although immunotherapy has gained much attention in the past few years, only some patients effectively respond, which deserves further exploration.

Genetic and epigenetic alterations are major hallmarks of cancers [6, 7]. Epigenetics refers to heritable alterations in gene expression without mediating DNA sequence changes, including DNA or RNA methylation, histone modification, and non-coding RNA (ncRNA). Among them, ncRNAs are transcribed from the genome and exert functions at the RNA level in mammals. Studies have shown that dysregulated ncRNAs are frequently found in cancers, especially microRNAs (miRNAs), long non-coding RNAs (lncRNAs), and circular RNAs (circRNAs) [8]. lncRNAs that are longer than 200 nucleotides are essential regulators of many biological processes, including gene expression, a decoy for transcription factors, and competing endogenous RNAs (ceRNAs) [9, 10]. A class of endogenous, small non-coding RNA molecules constituting 18–25 nucleotides is known as microRNAs (miRNAs). They can bind target messenger RNAs (mRNAs) in their 3′-untranslated regions (3′-UTRs), degrading them or inhibiting their translation, thus having a negative effect on the posttranscriptional regulation of gene expression. Studies have shown that lncRNAs can function as competitive endogenous RNAs (ceRNAs) for sponging miRNAs to destabilize mRNAs. This mechanism inhibits mRNA translation and impacts the body's physiological processes, which ultimately regulate gene expression [11, 12]. Mi et al. [10] demonstrated that high levels of the lncRNA AFAP1-AS1 could act as a molecular sponge of miR-26a and target ATF2, thus affecting the invasion and metastasis of esophageal carcinoma. Liu et al. [13] illustrated that SLC2A1-AS1 sponging miR-378a-3p resulted in Glut1 overexpression in esophageal carcinoma cells, promoting poor prognosis in esophageal carcinoma patients. However, previous studies have mainly focused on a single gene with limited predictive power; it is more meaningful to discover a cluster of genes and construct a multi-index and multimolecular model.

Notably, the deposition of ncRNAs is dynamic, allowing rapid responses to environmental events [14]. For instance, transcriptome analysis of KSHV-infected primary endothelial cells and B cell lines identified that human circRNAs had different responses to infection [15]. It has been well-accepted that genetics and lifestyles contribute to the entire cancer process, ranging from cancer etiology, prevention, early detection, diagnosis, and treatment to prognosis. A study by Gang et al. [16] identified that H19, a lncRNA involved in invasion and proliferation, was overexpressed and linked to pathological tumor size in smoking ESCC patients compared with non-smoking patients. Cigarette-induced miR-25-3p excessive maturation could promote pancreatic cancer progression [17]. However, the interplay between lifestyles and epigenetics in cancers has not been well studied. Previous studies have not systematically characterized the expression of smoking-related ncRNAs in esophageal carcinoma. In this study, we comprehensively explored the impacts of heavy smoking on the prognosis of esophageal carcinoma, the underlying alteration of ncRNA, and its correlation with the immune microenvironment based on the TCGA database. We profiled smoking-related ncRNAs, constructed an RNA regulatory network, explored the underlying molecular mechanism, and constructed a smoking-related risk score that revealed that tobacco smoking could suppress tumor immunity via the ncRNA mechanism.

## Materials and methods

### Datasets and differential expression analysis

The available RNA expression datasets and corresponding clinical characteristics of patients with esophageal carcinoma were obtained from TCGA. Specifically, the raw count expression profiles were retrieved from the TCGA database (https://portal.gdc.cancer.gov/), including 11 normal tissues and 160 tumorous tissues for lncRNA and mRNA, as well as 13 normal and 185 tumorous tissues for miRNA. Clinical data were acquired using “TCGAbiolinks”, composed of demographic characteristics (age, gender, BMI, smoking, and alcohol history), survival information (vital status, days to the last follow-up, and days to death), and clinical stage. Non-smoking was referred to as a lifelong non-smoker and a current reformed smoker for greater than 15 years. Heavy smokers (hereafter named smokers) were defined as those who smoked at least 20 pack-years among current smokers or current reformed smokers who smoked for less than or equal to 15 years.

The “edgeR” package was utilized for differential ncRNA expression analyses, with a filter of log |Fold change|> 1 and FDR < 0.05. These level-3 datasets are open-access and publicly available; thus, no approval was needed.

### Construction of the smoking-related regulatory network

For the smoking-related ceRNA regulatory network, we introduced the lncRNA‒miRNA and miRNA‒mRNA intersections. Specifically, the miRcode database (http://www.mircode.org/index.php) was applied to predict the target miRNA for lncRNA. Then, the overlapping miRNAs between the predicted miRNAs and the expressed miRNAs in esophageal carcinoma were candidates for lncRNA‒miRNA pairs. Next, the miRNA‒mRNA pairs (the abovementioned candidate miRNA and smoking-related miRNA) were predicted by miRTarBase (miRTarBase: the experimentally validated microRNA-target interactions database (cuhk.edu.cn)), miRDB (http://mirdb.org/), and Targetscan (http://www.targetscan.org/vert_72/). Pairs that were simultaneously predicted by all three methods were selected. Then, the overlapping ones between these predicted messenger RNAs (mRNAs) and the differentially expressed mRNAs in esophageal carcinoma were candidates for miRNA‒mRNA pairs. Finally, the smoking-related lncRNA‒miRNA‒mRNA axis was visualized by Cytoscape 3.7.1 software (https://cytoscape.org/).

### Functional and pathway enrichment analyses

To illustrate the functional annotations in smoking-related ncRNAs, we performed gene ontology (GO) analysis and Kyoto Encyclopedia of Genes and Genomes (KEGG) pathway enrichment analyses [18] using the “clusterProfiler” package with a cutoff of *P*-value < 0.05. Visualization of GO and KEGG results was realized by the “ggplot2” package.

### Identification and validation of the smoking-related prognostic signature

To investigate the smoking-related prognostic implication in esophageal carcinoma, we constructed a smoking-related molecular signature. In brief, univariate Cox regression analysis was first used to screen candidate mRNAs with a threshold of *P*-value < 0.1. Then, the least absolute shrinkage and selection operator (LASSO) Cox regression analysis was applied for signature selection and shrinkage using the “glmnet” package, with the parameter “maxit = 10,000”. The expression of smoking-related mRNAs was a candidate independent variable, and the overall survival time and status of patients with esophageal carcinoma were defined as the response variables. A smoking-related risk score was derived from the prediction model. All patients were categorized into high- or low-risk subgroups based on the median value of the score. Furthermore, the receiver operating characteristic (ROC) curve was utilized to evaluate the efficiency of the smoking-related prognostic risk score. The ROC curve was plotted, and the area under the curve (AUC) was calculated by the “survivalROC” package.

### Single sample gene set enrichment analysis (ssGSEA) and immune cell infiltration analysis

We employed single sample gene set enrichment analysis (ssGSEA) to explore the effects of smoking on the immune microenvironment. Namely, we compared the infiltration degree of 23 immune cell types between smoking-associated low-risk and high-risk groups. The relative abundance of each immune cell type was calculated by an enrichment score, which was normalized into a range from 0 to 1. Then, the biosimilarity of the infiltrating immune cells was evaluated by a “Gaussian” fitting model. The correlation analysis explored the association between smoking-related risk scores and immune cell types.

### Statistical analysis

Categorical variables are reported as frequencies and percentages. The Wilcoxon signed ranked test was adopted for comparison between normal and esophageal carcinoma groups. The hazard ratio (HR) and 95% confidence interval (CI), which were calculated by the Cox regression model, were used to estimate the strength of the association. We applied LASSO Cox regression analysis to screen mRNAs and extracted significant ones and their coefficients. The expression of smoking-related mRNAs was defined as an independent variable, and the overall survival time and status of patients with esophageal carcinoma were defined as the response variables to establish a smoking-related molecular prediction model. The survival analysis was performed by Kaplan‒Meier survival analysis with a log-rank test. ROC curves and AUCs were utilized to evaluate the efficiency of the smoking-related prognostic risk score. All statistical analyses were generated using R software, version 3.7.1 (https://www.r-project.org/), using the “edge”, “clusterProfiler”, “ggplot2”, “glmnet”, “survivalROC”, “survival”, and “forestplot” packages. A *P*-value less than 0.05 was considered statistically significant.

## Results

### Effect of heavy smoking on the prognosis of esophageal carcinoma

**Fig. 1:**
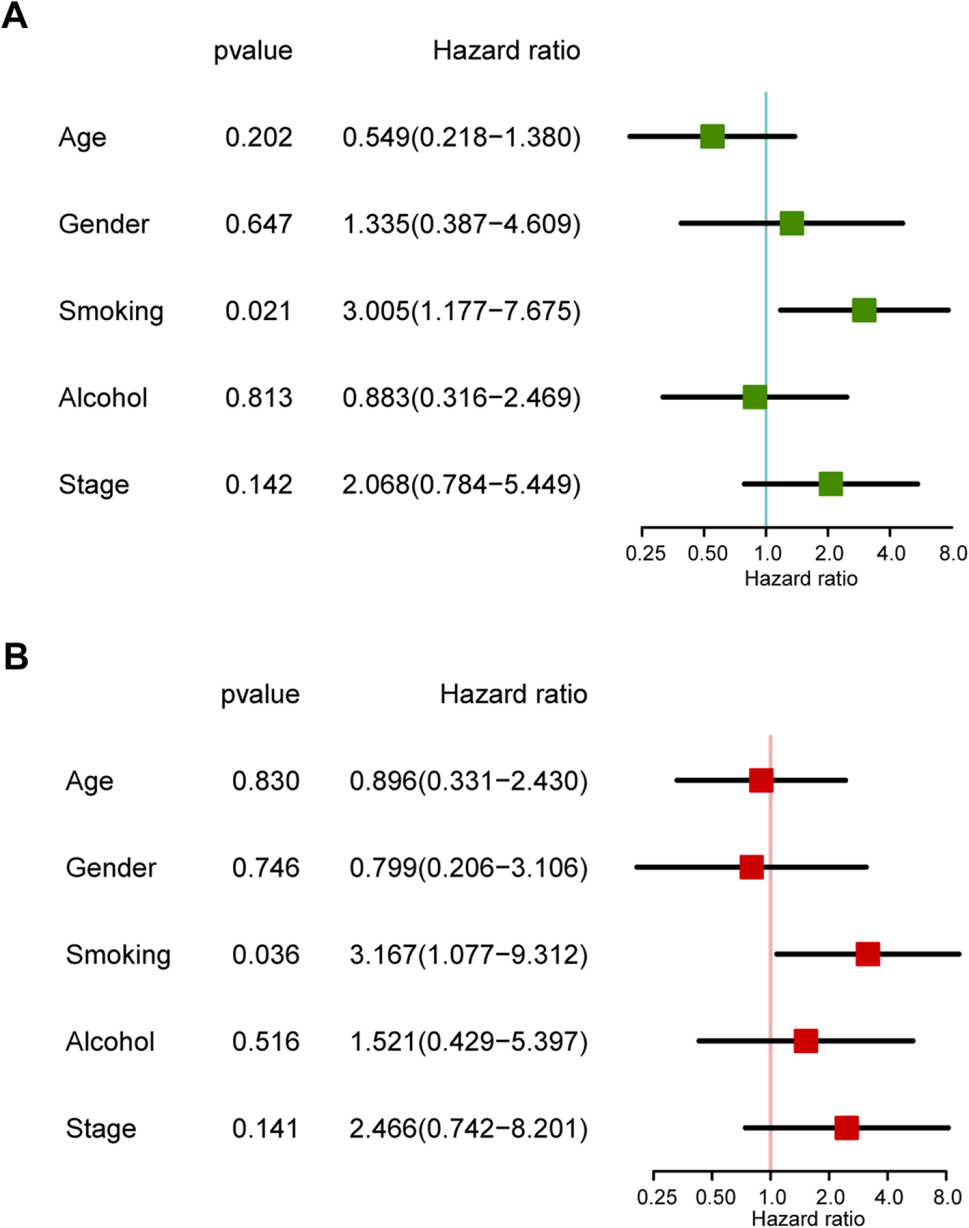
The association between tobacco smoking and overall survival in patients with esophageal carcinoma. **A** Univariate Cox regression analysis. **B** Multivariate Cox regression analysis

Most patients with esophageal carcinoma documented complete information on smoking (86.16%, 137/159) and alcohol consumption (98.11%, 156/159). After removing individuals who had missing survival time, clinicopathological information, tobacco smoking, and alcohol consumption data, we included 85 patients in the survival analysis. In the univariate Cox regression model, heavy smoking was significantly associated with the poor prognosis of patients with esophageal carcinoma, with an HR of 3.005 (95% CI: 1.777–7.675). No significant association was observed for alcohol consumption (HR: 0.883, 95% CI: 0.316–2.469) (Fig. 1A). In the multivariate Cox regression model, smoking was an independent risk factor for overall survival, with an HR of 3.167 (95% CI: 1.077–9.312) (Fig. 1B).

### Identification of smoking-related ncRNAs in esophageal cancer

**Fig. 2:**
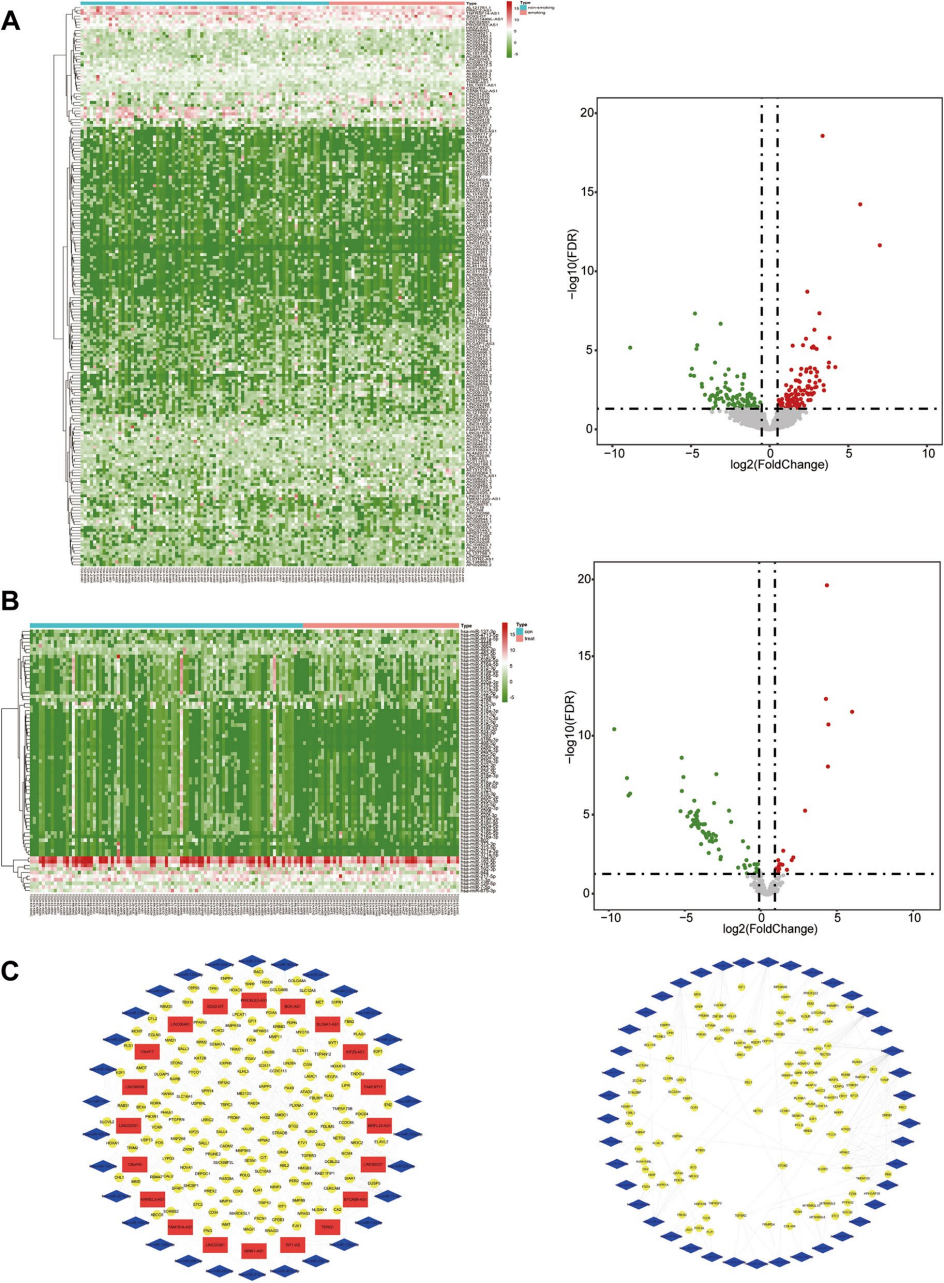
Identification of smoking-related ncRNAs in patients with esophageal carcinoma. **A** Heatmap and volcano plot showing the smoking-related differentially expressed lncRNAs in patients with esophageal carcinoma. **B** Heatmap and volcano plot showing the smoking-related differentially expressed miRNAs in patients with esophageal carcinoma. **C** The smoking-related ceRNA and miRNA regulatory network in esophageal carcinoma

To determine the expression patterns of ncRNAs modified by smoking in esophageal carcinoma, we profiled the differentially expressed ncRNAs between non-smokers and smokers. Of these, 13,162 RNAs were successfully mapped into lncRNAs, and 2068 RNAs were mapped into miRNAs. A total of 195 differentially expressed lncRNAs (DElncRNAs), including 108 upregulated and 87 downregulated lncRNAs, and 73 differentially expressed miRNAs (DEmiRNAs), including 10 upregulated and 63 downregulated miRNAs, were identified in smokers (Fig. 2A and B). Among them, AC092484.1 and AC021713.1 were identified as the most upregulated and downregulated lncRNAs, respectively. Meanwhile, hsa-miR-216b-5p and hsa-miR-372-3p were defined as the most upregulated and downregulated lncRNAs, respectively.

### Construction of smoking-related regulatory network and functional annotation

Considering the critical function of miRNAs in inhibiting target mRNAs and the mechanism of well-recognized ceRNAs, we constructed a smoking-related regulatory network in esophageal carcinoma. We first identified 3139 differentially expressed mRNAs in esophageal carcinoma, including 1399 upregulated and 1740 downregulated mRNAs (Supplemental Fig. 1). For the smoking-related ceRNA regulatory network, we predicted lncRNA‒miRNA and miRNA‒mRNA intersections. As a result, we identified 83 lncRNA‒miRNA pairs and 195 miRNA‒mRNA pairs, composed of 11 lncRNAs, 28 miRNAs, and 138 mRNAs (Fig. 2C). Additionally, we determined 186 smoking-related miRNA‒mRNA pairs based on smoking-related miRNAs, including 41 miRNAs and 125 mRNAs (Fig. 2C).

**Fig. 3:**
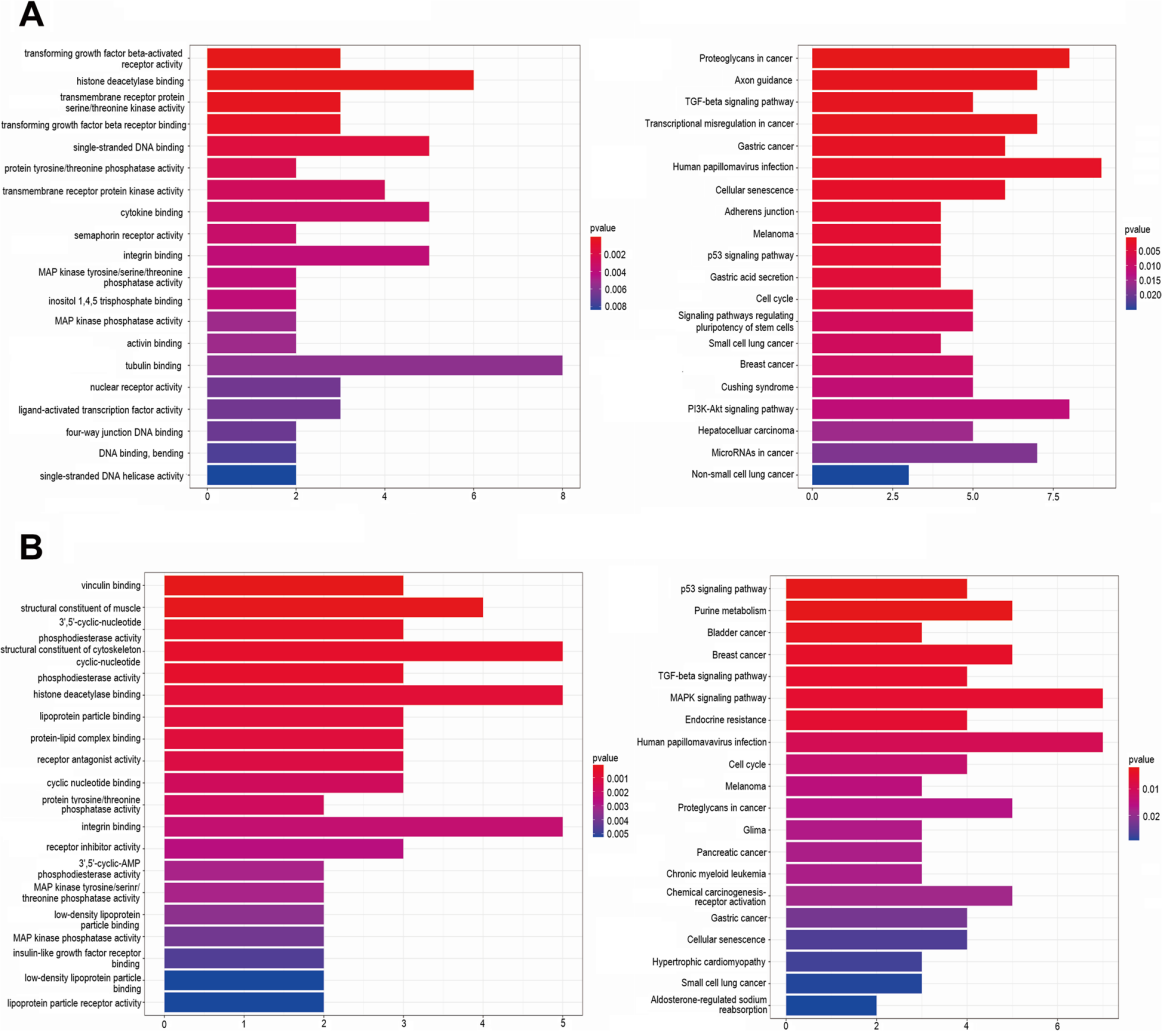
Functional prediction of smoking-related ncRNAs in patients with esophageal carcinoma. **A** GO and KEGG analyses of smoking-related lncRNAs in esophageal carcinoma. **B** GO and KEGG analyses of smoking-related miRNAs in esophageal carcinoma

To better understand the underlying functions of smoking, we conducted GO and KEGG pathway enrichment analyses. For the target mRNAs of smoking-related lncRNAs, a total of 45 GO terms and 35 KEGG pathways were enriched. In particular, several cancer-related pathways were observed, such as proteoglycans in cancer, the TGF-β signaling pathway, the p53 signaling pathway, and the PI3K-Akt signaling pathway (Fig. 3A). For the target mRNAs of smoking-related miRNAs, a total of 36 GO terms and 25 KEGG pathways were enriched, including the p53 signaling pathway, purine metabolism, TGF-β signaling pathway, and MAPK signaling pathway (Fig. 3B).

### Generation of smoking-related prognostic signature and survival analysis

**Fig. 4:**
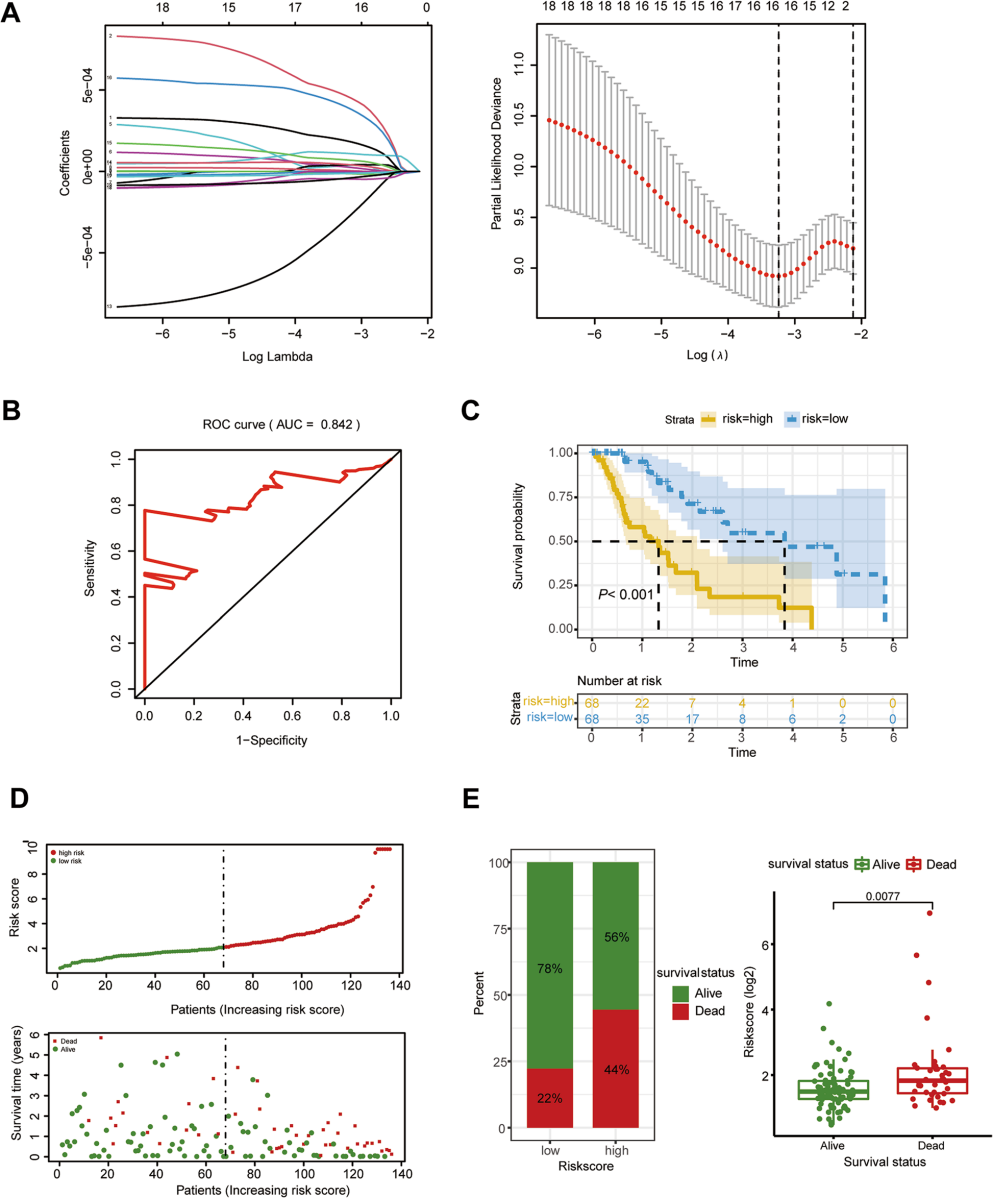
Construction of a smoking-related prognostic model in patients with esophageal carcinoma. **A** The LASSO Cox regression analysis was constructed from 19 candidates derived from univariate Cox regression, and the tuning parameter (λ) was calculated based on the partial likelihood deviance with tenfold cross-validation. Then, an optimal log λ value is indicated by the dotted line in the plot. **B** The ROC curve for validation of the efficiency of the smoking-related risk score. **C** Kaplan‒Meier analysis with log-rank test for survival analysis in smoking-related subgroups. The median was defined as the cutoff value. **D** The risk plot of survival time and risk score. **E** The proportion of survival status in smoking-related subgroups

In terms of the significance of smoking and related ncRNAs in esophageal carcinoma, we further constructed a smoking-related prognostic signature based on LASSO Cox regression analysis. In total, we obtained 208 target mRNAs of smoking-related ncRNAs. Nineteen candidates were first screened by univariate Cox regression analysis, followed by 16 smoking-related prognostic signatures recognized by Lasso Cox regression, and then a smoking-related risk score was generated (Fig. 4A). The risk score was calculated by considering EGFR2, RIMS3, CADM2, HMGB3, E2F1, PLAU, FYCO1, TGFBR2, KLHL15, SNCG, ATAD2, KIAA1549L, HHIP, CYBRD1, CELF2, and FBLN2. The ROC curve revealed that such a risk score could well distinguish patients into two subgroups, with an AUC of 0.842 (Fig. 4B).

**Fig. 5:**
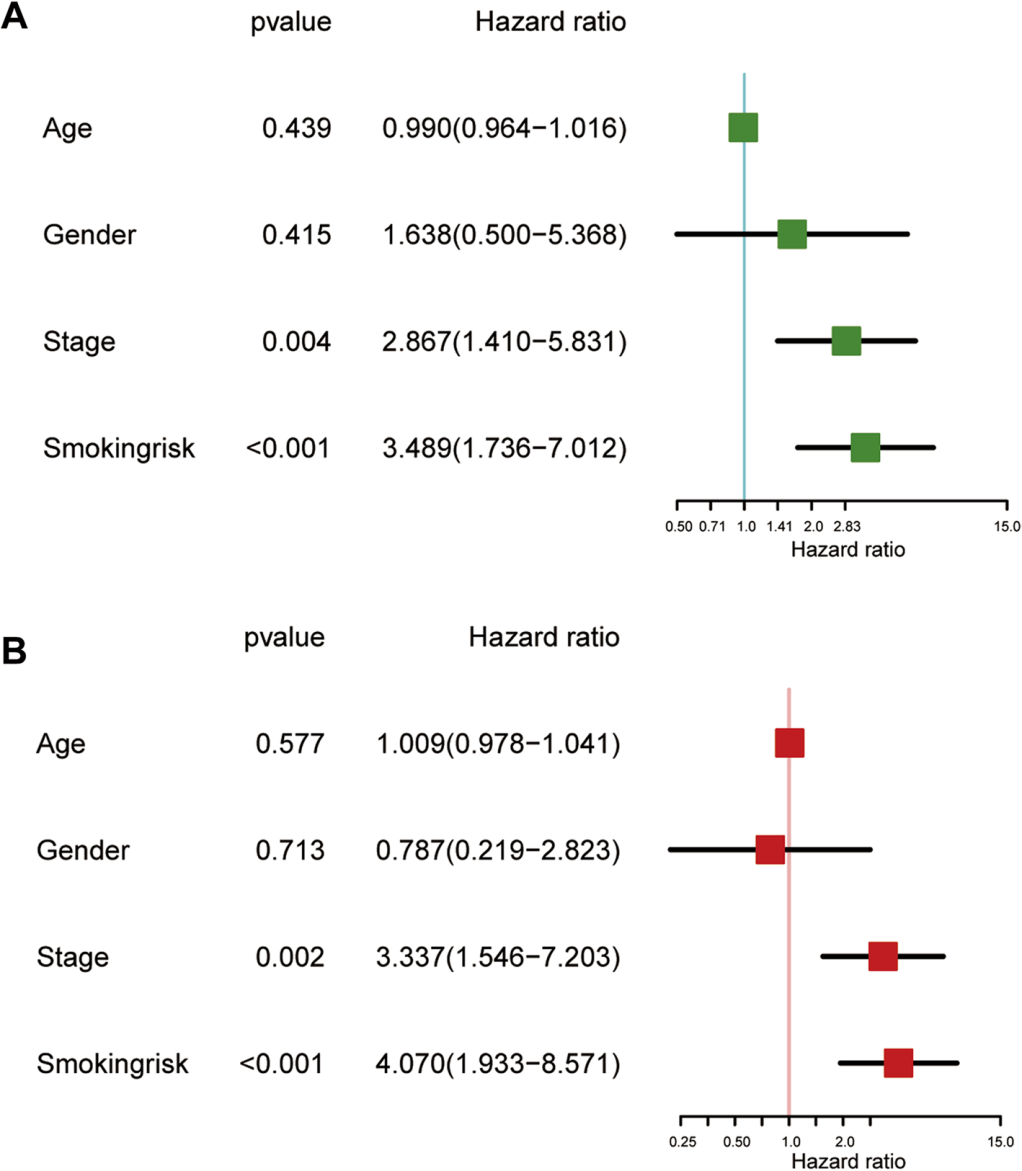
The association between smoking-related ncRNAs and overall survival in patients with esophageal carcinoma. **A** Univariate Cox regression analysis. **B** Multivariate Cox regression analysis

Subsequently, we stratified patients into high- and low-risk subgroups based on the median value of the risk score. Patients with a low-risk score had a better overall survival (Fig. 4C). As indicated in Fig. 4D, patients with a high-risk score exhibited a higher risk of death. The proportion of survivors in the low-risk group was higher than that in the high-risk group, and survivors exhibited a lower risk score than that of deaths (Fig. 4E). The univariate and multivariate Cox regression analyses suggested that smoking-related risk factors were linked to a poor prognosis, with a crude HR of 3.489 (95% CI: 1.736–7.012) and an adjusted HR of 4.070 (95% CI: 1.933–8.571) (Fig. 5A and B).

**Fig. 6:**
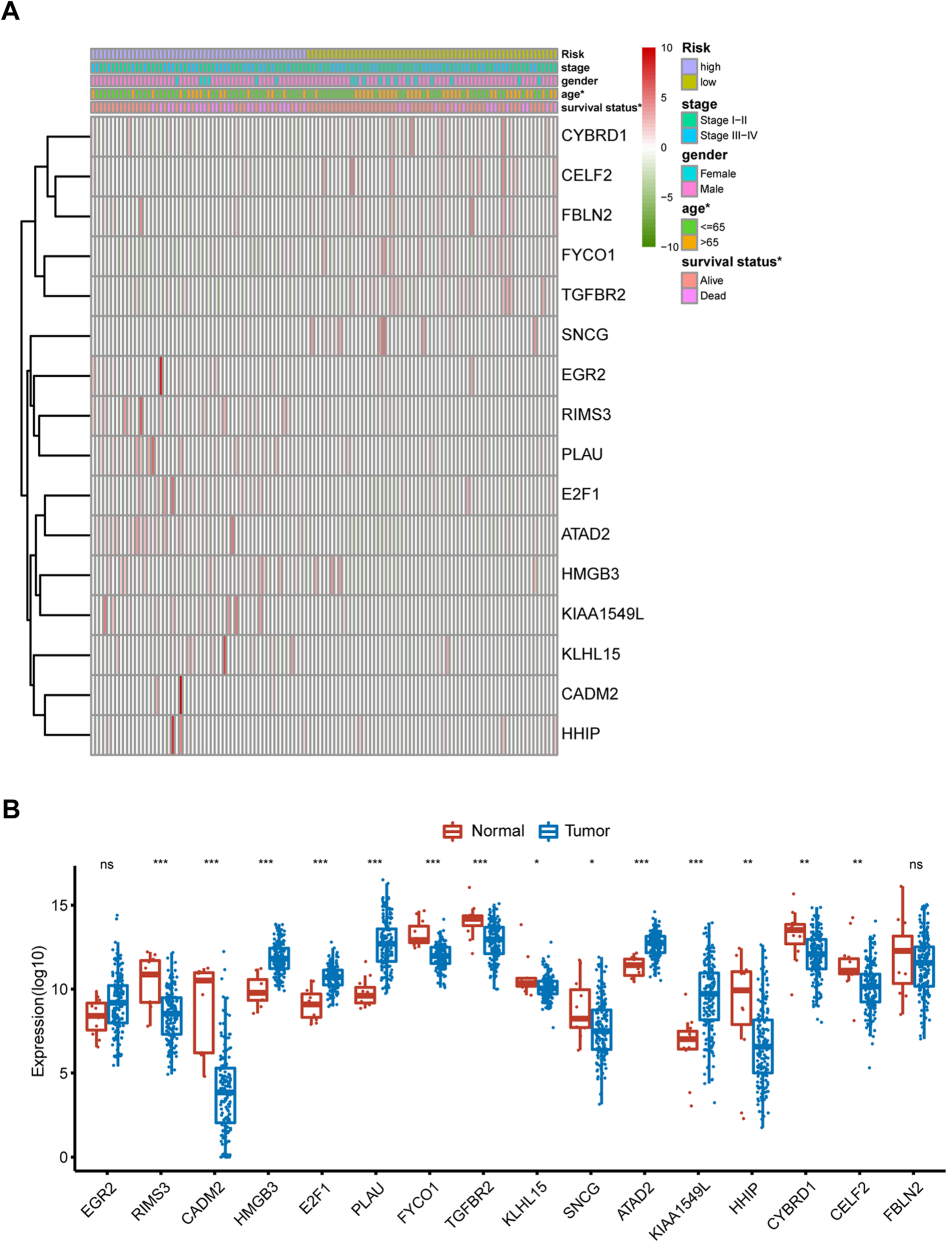
The expression patterns of smoking-related signatures in distinct subgroups. **A** A heatmap visualizing the expression of the 16 smoking-related signatures in distinct subgroups. **B** Comparison of the 16 smoking-related signatures between normal controls and esophageal carcinoma patients

We also illustrated the expression patterns of each signature among distinct clinical characteristics (Fig. 6A). Comparison of these signatures between normal and tumor samples indicated that HMGB3, E2F1, PLAU, ATAD2, and KIAA1549L were highly expressed in tumor tissues, while RIMS3, CADM2, FYCO1, TGFBR2, KLHL15, SNCG, HHIP, CYBRD1, and CELF2 were downregulated (Fig. 6B).

### Association between smoking and immune cell infiltration

**Fig. 7:**
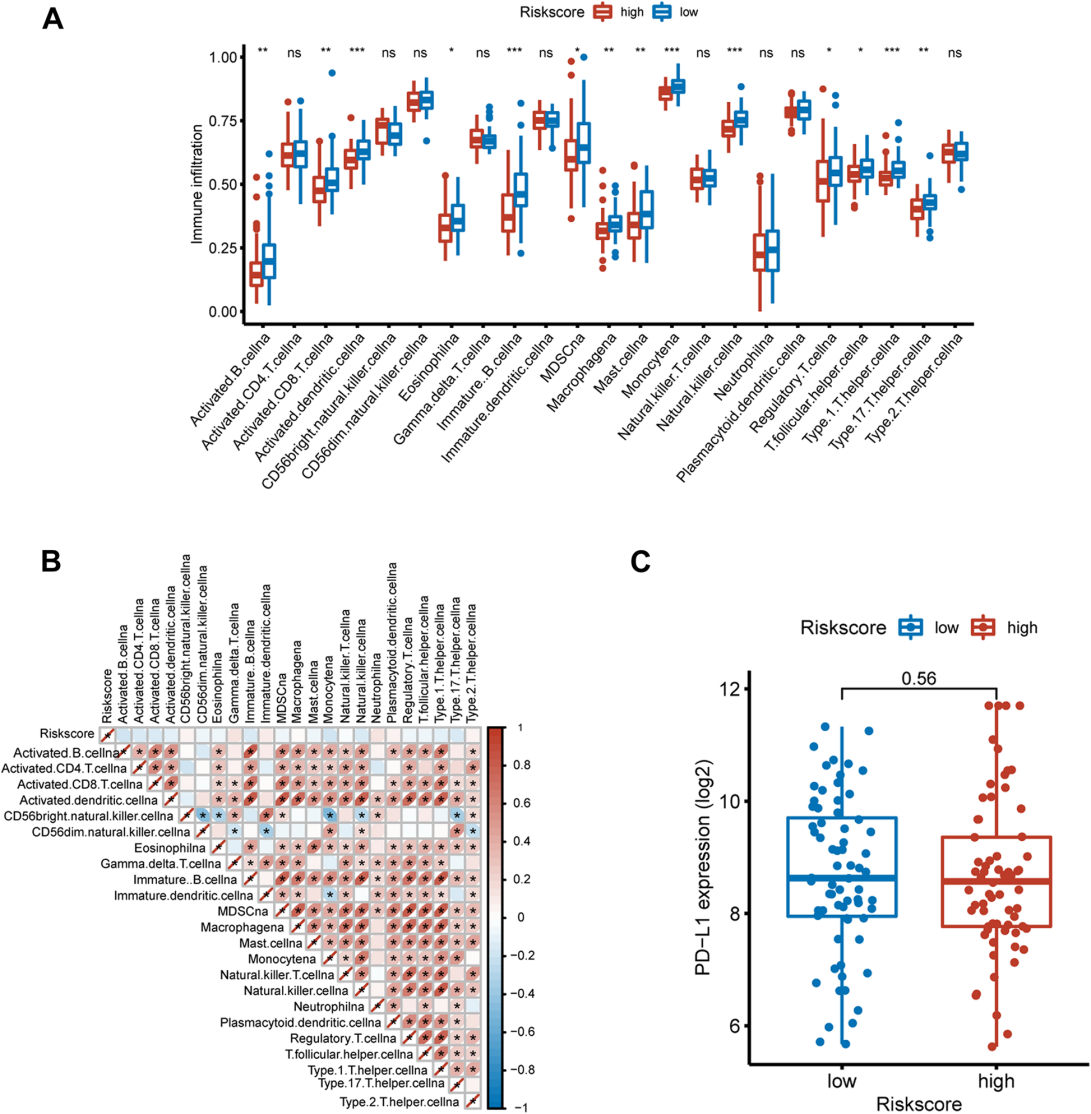
The immune landscape of immune cell types in smoking-stratified groups. **A** Comparison of the fractions of immune cells between smoking-related high- and low-risk subgroups. **B** Correlation between the fractions of immune cells and smoking-related risk score. **C** Comparison of PD-L1 expression in smoking-related high- and low-risk subgroups

To assess the impact of smoking on immune status in patients with esophageal carcinoma, we used ssGSEA to calculate the immune infiltration for each patient. Interestingly, we found that several immune cells were differentially enriched in smoking-related subgroups. Among them, 14 out of 23 immune cell types were downregulated in the high-risk group, including activated B cells, CD8 + T cells, dendritic cells, eosinophils, immature B cells, MDSCs, monocytes, natural killer cells, regulatory T cells, T follicular helper cells, type 1 helper cells, type 17 helper cells and type 2 helper cells (Fig. 7A). We then evaluated the correlation between the smoking-related risk score and each type of immune cell, and no significant association was observed (Fig. 7B). Additionally, the expression of PD-L1 in the low- and high-risk groups was comparable (Fig. 7C).

## Discussion

Tobacco smoking is a well-known risk factor for the occurrence and progression of many human cancers, including lung, bladder, head and neck, pancreatic and esophageal cancer [19–21]. To date, over 70 types of tobacco carcinogens have been described, including polycyclic aromatic hydrocarbons, nitrosamines, aromatic amines, aldehydes, various hydrocarbons, and other organics [22]. Recently, the altered RNA modifications induced by recurrent chemical stimulation have received much attention, especially for ncRNA in cancers. In this study, we comprehensively explored the impacts of tobacco smoking on prognosis, the underlying alteration of ncRNA, and its correlation with the immune microenvironment in esophageal carcinoma based on the TCGA database.

Historically, Doll and Hill's research exerted a crucial role in the initial understanding of tobacco-related risks and laid the foundation for future research in the 1950s [23]. Although substantial efforts have been devoted to this limitation, tobacco smoking remains a significant problem, with an estimated 1.1 billion smokers across the 195 countries and territories that are assessed by the global burden of diseases, injuries, and risk factors [24]. Hence, it is necessary to evaluate the effect of tobacco smoking on human diseases, including cancers. A multivariable Mendelian randomization analysis demonstrated strong evidence for an independent causal effect of smoking on oral/oropharyngeal cancer [25]. Yang et al. found that smoking at diagnosis increased mortality risk compared with reformed smokers in the head and neck, lung squamous cell carcinoma, and pancreatic adenocarcinoma [26]. In this study, we found that heavy smoking was an independent risk factor for overall survival in esophageal carcinoma, with an HR of 3.167. Therefore, it is essential to further elucidate the smoking-related molecular mechanism.

The ncRNA modification is dynamic and thereby allows rapid responses to environmental events. Wang et al. revealed that PM2.5 induced a series of altered miRNAs in human endothelial cells [27]. Moreover, 27 lncRNAs and 32 miRNAs were differentially expressed between patients with or without *H. pylori* infection [28]. To explore whether tobacco smoking can modify the expression patterns of ncRNAs in esophageal carcinoma, we profiled smoking-related ncRNAs. A total of 195 differentially expressed lncRNAs and 73 differentially expressed miRNAs were identified. Of these, hsa-miR-372-3p has been previously found to inhibit the proliferation and metastasis of osteosarcoma cells [29]. Recently, ceRNA has promoted our understanding of cancers. We then constructed a smoking-related RNA regulatory network to exhibit the potential molecular regulation. Moreover, functional annotation revealed that several cancer-related pathways were enriched. Additionally, we generated a smoking-related risk score and found that patients with low scores had a better prognosis than those with high scores. These findings indicated that smoking could participate in the occurrence or progression of esophageal carcinoma by modifying ncRNA patterns.

Previous studies have demonstrated that tobacco smoking profoundly impacts immunity [30]. For instance, nicotine, one of the primary constituents of tobacco smoking, was reported to modulate molecules of the innate immune response in epithelial cells and macrophages during infection with M. tuberculosis [31]. Matthew C Madison et al. found that electronic cigarettes could disrupt lung lipid homeostasis and innate immunity independent of nicotine [32]. Furthermore, m6A-related lncRNAs could affect the prognosis and tumor immune microenvironment in patients with lung adenocarcinoma. To investigate whether tobacco smoking could affect tumor immunity via ncRNAs, we assessed the association between 23 immune cell types and smoking-related risk scores. As a result, 14/23 immune cells were downregulated in patients with high risk, indicating that tobacco smoking could suppress tumor immunity via ncRNA modification. However, the smoking-related risk score presented no correlation with immune cells or the expression of PD-L1.

It should be noted that our study has several limitations. First, our research is a single-center design based on the TCGA database, and external validation seems more meaningful. Second, our current findings are derived from bioinformatic analysis, and further experimental research is needed.

## Conclusion

In this study, we identified a series of smoking-related ncRNAs and constructed an RNA regulatory network, providing a landscape of smoking-related molecular mechanisms. We further generated a smoking-related risk score and revealed that tobacco smoking could suppress tumor immunity via ncRNA alterations.

## Supplementary Information

**Additional file 1: Figure S1.** The association between tobacco smoking and overall survival in patients with esophageal carcinoma.

## Data Availability

The datasets generated during and/or analyzed during the current study are available in the TCGA repository, [https://portal.gdc.cancer.gov/].

## Abbreviations

ncRNA: Non-coding RNA
lncRNA: Long non-coding RNA
mRNA: Messenger RNA
circRNA: Circular RNA
miRNA: Micro RNA
ceRNA: Competing endogenous RNA
TCGA: The Cancer Genome Atlas
HR: Hazard ratio
CI: Confidence interval
GO: Gene Ontology
KEGG: Kyoto Encyclopedia of Genes and Genomes
LASSO: Least absolute shrinkage and selection operator
ESCC: Esophageal squamous cell carcinoma
EAC: Esophageal adenocarcinoma
ROC: Receiver operating characteristic
AUC: Area under the curve
ssGSEA: Single sample gene set enrichment analysis
DElncRNA: Differentially expressed lncRNA
DEmiRNA: Differentially expressed miRNA
