# Supplementary material for: Impacts of heavy smoking on non-coding RNA expression for patients with esophageal carcinoma
Source: BMC Med Genomics. 2023 Jul 5;16:157. doi: 10.1186/s12920-023-01574-z (PMC10324184; doi:10.1186/s12920-023-01574-z)

**Supplementary materials**

**Figure S1 The association between tobacco smoking and overall survival in patients with esophageal carcinoma.**

A volcano is showing the differentially expressed mRNAs in patients with esophageal carcinoma.


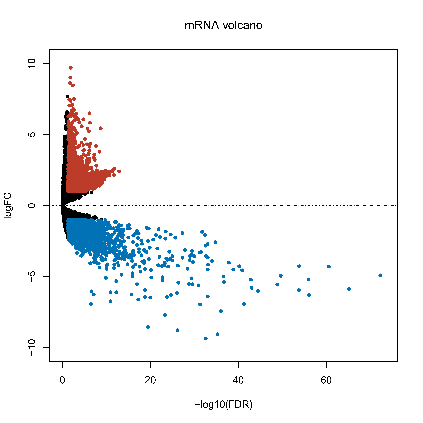

Supplement: Supplementary file 1 — Additional file 1: Figure S1. The association between tobacco smoking and overall survival in patients with esophageal carcinoma. [file 12920_2023_1574_MOESM1_ESM.docx]
